# Antimicrobial Peptides: A Promising Alternative to Conventional Antimicrobials for Combating Polymicrobial Biofilms

**DOI:** 10.1002/advs.202410893

**Published:** 2024-11-12

**Authors:** Cesar Augusto Roque‐Borda, Laura Maria Duran Gleriani Primo, Kaila Petronila Medina‐Alarcón, Isabella C. Campos, Camila de Fátima Nascimento, Mauro M. S. Saraiva, Angelo Berchieri Junior, Ana Marisa Fusco‐Almeida, Maria José Soares Mendes‐Giannini, João Perdigão, Fernando Rogério Pavan, Fernando Albericio

**Affiliations:** ^1^ Department of Biological Sciences School of Pharmaceutical Sciences Universidade Estadual Paulista (UNESP) Araraquara Sao Paulo 14800‐903 Brazil; ^2^ iMed.ULisboa–Institute for Medicines Research Faculty of Pharmacy University of Lisbon Lisbon 1649004 Portugal; ^3^ Vicerrectorado de Investigación Universidad Católica de Santa María Arequipa 04000 Peru; ^4^ Department of Clinical Analysis School of Pharmaceutical Sciences Universidade Estadual Paulista (UNESP) Araraquara Sao Paulo 14800‐903 Brazil; ^5^ São Paulo State University (UNESP) School of Agricultural and Veterinarian Sciences Jaboticabal Sao Paulo 14884‐900 Brazil; ^6^ Peptide Science Laboratory School of Chemistry and Physics University of KwaZulu‐Natal Durban 4001 South Africa; ^7^ CIBER‐BBN Networking Centre on Bioengineering Biomaterials and Nanomedicine and Department of Organic Chemistry University of Barcelona Barcelona 08028 Spain

**Keywords:** antimicrobial alternatives, biofilms, drug discovery, polymicrobial interactions

## Abstract

Polymicrobial biofilms adhere to surfaces and enhance pathogen resistance to conventional treatments, significantly contributing to chronic infections in the respiratory tract, oral cavity, chronic wounds, and on medical devices. This review examines antimicrobial peptides (AMPs) as a promising alternative to traditional antibiotics for treating biofilm‐associated infections. AMPs, which can be produced as part of the innate immune response or synthesized therapeutically, have broad‐spectrum antimicrobial activity, often disrupting microbial cell membranes and causing cell death. Many specifically target negatively charged bacterial membranes, unlike host cell membranes. Research shows AMPs effectively inhibit and disrupt polymicrobial biofilms and can enhance conventional antibiotics' efficacy. Preclinical and clinical research is advancing, with animal studies and clinical trials showing promise against multidrug‐resistant bacteria and fungi. Numerous patents indicate increasing interest in AMPs. However, challenges such as peptide stability, potential cytotoxicity, and high production costs must be addressed. Ongoing research focuses on optimizing AMP structures, enhancing stability, and developing cost‐effective production methods. In summary, AMPs offer a novel approach to combating biofilm‐associated infections, with their unique mechanisms and synergistic potential with existing antibiotics positioning them as promising candidates for future treatments.

## Graphical abstract

Antimicrobial peptides (AMPs) are promising alternatives to conventional treatments for polymicrobial biofilms, which increase pathogen resistance. AMPs disrupt biofilm extracellular matrices and microbial membranes, inhibiting biofilm formation and enhancing antibiotic efficacy. Current research addresses challenges like stability and production costs to optimize clinical applications, highlighting AMPs' innovative potential in treating biofilm‐associated infections.

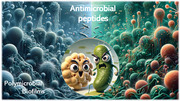

## 1 Introduction

**Scheme 1:**
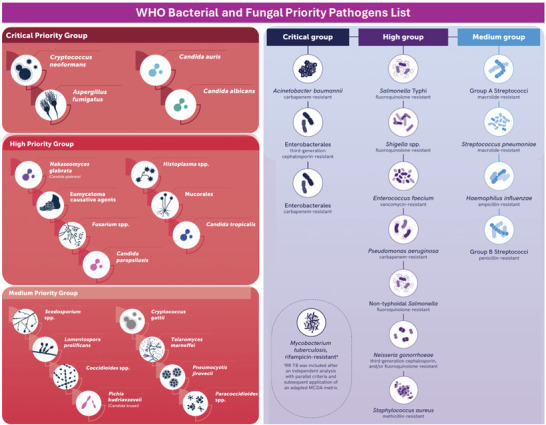
List of priority fungi (left) and bacteria (right) launched by the WHO in 2022 and 2024, respectively. Reproduced (adapted) with permission from World Health Organization, Copyright 2022 and 2024, WHO.

Chronic diseases caused by microorganisms are on the rise, and with growing concern, the World Health Organization (WHO) released an updated list of priority bacteria in 2024 and, for the first time, a list of priority fungi in 2022.^[^ ^1^, ^2^ ^]^ This update was driven by the increasing threat of antimicrobial resistance, which undermines the effectiveness of current treatments and poses a significant risk to global health. The update included various significant changes, one notable addition was rifampicin‐resistant *Mycobacterium tuberculosis* (*M*. *tuberculosis*), which was added to the critical priority list based on Multicriteria Decision Analysis. This bacterium is known for causing tuberculosis, a severe and often deadly respiratory disease and its inclusion highlights the urgent need for new treatments and strategies to combat its resistance. Interestingly, *Pseudomonas aeruginosa* (*P*. *aeruginosa*) resistant to carbapenems was moved from the critical priority group to the high priority group but is important to note that this bacterium remains a significant concern, particularly in healthcare settings where it can cause severe infections in vulnerable patients (**Scheme** **1**).^[^ ^3^ ^]^

The increasing prevalence of multiple bacterial infections is compounded by the presence of weakened immune systems, which can lead to the acquisition of other opportunistic pathogens.^[^ ^4^ ^]^ These pathogens exploit the compromised immune defenses of the host, leading to further complications and spreading more easily. This interplay between different microorganisms complicates treatment and highlights the need for a comprehensive approach to infection control and antimicrobial stewardship.^[^ ^5^ ^]^ Moreover, bacteria and fungi can work together to enhance their survival in the presence of antimicrobial agents. This cooperation is often facilitated through the production of protective extracellular molecules, such as biofilms. Biofilms are complex communities of microorganisms that adhere to surfaces and are encased in a self‐produced matrix. This matrix protects the microorganisms from antimicrobial agents and the host's immune response, making infections difficult to eradicate.^[^ ^6^ ^]^ However, during infections, complex microbial communities adhere to surfaces and significantly contribute to chronic infections by enhancing pathogen resistance to conventional treatments. These multispecies communities produce a protection matrix called Polymicrobial biofilms. Therefore, polymicrobial biofilms are communities of microbial cells in which these cells are from multispecies.^[^ ^7^ ^]^ These biofilms are implicated in a wide range of infections, including those affecting the respiratory tract, oral cavity, chronic wounds, and medical devices, posing substantial clinical challenges. Traditional antibiotic treatments often fail against these resilient biofilms, necessitating the exploration of alternative therapeutic strategies.^[^ ^8^ ^]^

Antimicrobial peptides (AMPs) have emerged as a promising alternative to traditional antibiotics for treating biofilm‐associated infections. AMPs are small molecules produced as part of the innate immune response and exhibit broad‐spectrum antimicrobial activity.^[^ ^9^ ^]^ They can disrupt microbial cell membranes through various mechanisms, leading to cell lysis and death.^[^ ^10^ ^]^ This disruption is often due to their interaction with negatively charged bacterial membranes, which differ from the membranes of host cells. However, AMPs employ several mechanisms of action beyond membrane disruption. These include inhibition of cell wall synthesis, interference with nucleic acid synthesis, modulation of immune responses, and disruption of essential metabolic processes within microorganisms.^[^ ^11^ ^]^ Studies have demonstrated the efficacy of AMPs against polymicrobial biofilms, showing significant inhibition and disruption properties. In addition to their standalone capabilities, AMPs can enhance the effectiveness of conventional antibiotics, allowing for reduced dosages and potentially mitigating the development of antibiotic resistance.^[^ ^12^, ^13^ ^]^ AMPs offer a novel approach to combating polymicrobial biofilms in chronic infections. Their diverse mechanisms of action, broad‐spectrum activity, and synergistic potential with existing antibiotics make them promising candidates for addressing the challenges associated with biofilm‐associated infections. Here, we propose to debater the polymicrobial biofilms and how AMPs can be used as an alternative as conventional antimicrobials.

In prior reviews, the mechanisms by which AMPs inhibit biofilms were extensively discussed, focusing on their ability to disrupt biofilm matrix components like polysaccharides and proteins, prevent bacterial adhesion and initial biofilm formation, and penetrate mature biofilm structures to target embedded bacteria.^[^ ^14^, ^15^, ^16^, ^17^ ^]^ Despite these findings, most studies focus on specific AMP types or biofilm models, leaving considerable room for broader investigations across diverse AMP classes and biofilm conditions.^[^ ^18^ ^]^ Current literature reveals gaps, such as limited broad‐spectrum assessments of AMP efficacy across different biofilm‐forming bacteria, variability in experimental models that hinders comparability and relevance to realistic environments (e.g., polymicrobial or physiological fluid biofilms), and insufficient mechanistic insights into AMP activity under physiological conditions, particularly in complex environments like chronic wounds or lung infections.^[^ ^18^, ^19^ ^]^

This study aims to fill these gaps by evaluating a wider spectrum of AMPs against various biofilm‐forming pathogens, including those implicated in polymicrobial infections, and employing standardized and complex biofilm models that simulate physiological conditions for a more accurate assessment of AMP efficacy. Additionally, it investigates AMP mechanisms and resistance within biofilms under different conditions, such as high ionic strength environments, to develop strategies that enhance AMP stability and efficacy in clinical contexts. By providing comprehensive insights into AMP‐biofilm interactions, the study informs the development of novel AMP‐based therapies targeting biofilm‐associated infections, offering improved treatment options for persistent and drug‐resistant infections.

## 2 Biofilm Formation

Recently, microorganisms in the planktonic form have been associated with acute infections treatable with conventional antibiotics if diagnosed promptly. However, when associated with other microorganisms forming biofilms, provoke infections that often become intractable and chronic due to low‐grade inflammation,^[^ ^20^, ^21^ ^]^ as discussed before. Biofilm formation has been extensively investigated using in vitro models, focusing on surface colonization.^[^ ^20^, ^22^ ^]^ Attachment, facilitated by adhesins and EPS secretion, leads to irreversible cell attachment and biofilm maturation, featuring interconnected structures with channels for essential substance transport.^[^ ^23^ ^]^ EPS is a crucial adhesion framework and the main component of biofilms.^[^ ^24^, ^25^ ^]^ with its polysaccharide composition varying across bacterial and fungal groups. In *P. aeruginosa*, EPS primarily includes alginate, *Pel*, and *Psl*, with phosphoglucomutase enzymes aiding their synthesis.^[^ ^26^ ^]^ Extracellular proteins, such as the glucan‐binding protein (Gbp) in *S. mutans*, are vital for biofilm stability.^[^ ^27^ ^]^ During biofilm maturation, QS mechanisms regulate biomass and nutrient availability, ensuring structural integrity.^[^ ^28^, ^29^ ^]^ Gene regulation in biofilms depends on the microorganism species and their interactions, whether in monospecies or polymicrobial biofilms,^[^ ^30^ ^]^ hence the importance of QS mechanisms on this structure.

Polymicrobial biofilm formation (PMBF) involves interspecies, intergenic, and interkingdom interactions, characterized by a three‐dimensional exopolysaccharide matrix with multiple microbial species,^[^ ^31^ ^]^ These biofilms are composed of polysaccharides, nucleic acids, and proteins grouped as EPS matrices.^[^ ^32^, ^33^ ^]^ PMBF can arise from microbiota dysbiosis, with primary colonizers facilitating the transition from planktonic to sessile forms.^[^ ^34^, ^35^ ^]^ QS mediates microbial communication within and between species and across different kingdoms during PMBF development.^[^ ^36^, ^37^, ^38^ ^]^ Biofilm formation is a highly complex and very dynamic process that goes from the process of initial adhesion of planktonic cells to the substrate, with adhesion and formation of microcolonies, with the production of EPS which determines the assembly of the biofilm due to continuous production on site, promoting colonization and cell grouping from the adhesion phase ending in the dispersion of the biofilm where microorganisms look for new niches to colonize.^[^ ^33^, ^39^, ^40^ ^]^ Bacteria residing within biofilms exhibit distinct characteristics compared to free‐living planktonic cells, including altered physiology characterized by heightened resistance to antibiotics and the immunity system, facilitating the establishment of chronic and persistent infections.^[^ ^41^ ^]^ Various factors contribute to biofilm formation, including environmental conditions, nutrient levels, and temperature, with deviations from optimal ranges exerting adverse effects,^[^ ^42^ ^]^ pH levels influence surface cell hydrophobicity^[^ ^43^ ^]^ while ionic strength can impact biofilm formation.^[^ ^44^ ^]^ Additionally, factors affecting bacterial adhesion include cell surface properties such as hydrophobicity, flagellum and motility, surface roughness, and hydrodynamic conditions.^[^ ^45^ ^]^ Bacterial adhesion is directly influenced by environmental moisture, increased adhesion is noted in environments with higher humidity.^[^ ^46^ ^]^

It has been reported that on hydrophobic and rough surfaces the biofilm shows better adhesion.^[^ ^47^ ^]^ Promoting robust adherence to hydrophobic surfaces, whereas hydrophilic bacterial cells adhere to surfaces with similar hydrophilicity.^[^ ^48^ ^]^ Properties such as the presence of extracellular appendages like fimbriae and flagella, cell–cell interactions, and communication via EPS, polysaccharides, or proteins confer a competitive advantage for mixed formations. Thus, mitigating the risk of nosocomial pathogen transmission requires influencing these factors to regulate their formation and thereby prevent infection establishment, focusing primarily on methods to eradicate the biofilm.^[^ ^15^, ^49^, ^50^ ^]^

## 3 Polymicrobial Biofilms

Approximately 80% of chronic human infections, such as otitis media, diabetic foot ulcers, oral, wound, and respiratory illnesses are associated with bacterial biofilm formation.^[^ ^51^, ^52^ ^]^ Initially observed in cystic fibrosis patients in 1973,^[^ ^53^, ^54^ ^]^ this condition now affects about 162,400 individuals globally.^[^ ^55^ ^]^ Common pathogens include *Staphylococcus aureus* (*S. aureus*), *P. aeruginosa*, and emerging challenges such as *Achromobacter xylosoxidans* (*A*. *xylosoxidans*), *Burkholderia cepacian* (*B. cepacia*), *Stenotrophomonas maltophilia* (*S*. *maltophilia*), and mycobacteria.^[^ ^56^ ^]^ Fungal biofilms on medical devices have also become significant nosocomial issues. Polymicrobial biofilms involving *P. aeruginosa* and *B. cepacia* were reported in cystic fibrosis patients in 1995; Hogan and Kolter described *Candida albicans* (*C*. *albicans*) and *S. aureus* interactions in mixed biofilms in 2002.^[^ ^57^ ^]^

The human oral cavity is a major site of polymicrobial biofilm, contributing to various oral diseases.^[^ ^58^ ^]^ Periodontitis, mainly caused by *Porphyromonas gingivalis* (*P*. *gingivalis*), has seen an 83.4% increase in global incidence from 1990 to 2019.^[^ ^59^, ^60^ ^]^ Dental caries often involve *Streptococcus mutans* (*S. mutans*) and other acidogenic bacteria, exacerbated by dietary sucrose.^[^ ^61^ ^]^ Additionally, patients with type 1 diabetes exhibit higher bacterial loads and dental decay rates due to reduced salivary flow and altered oral pH regulation.^[^ ^62^ ^]^ Other recurrent illnesses that affect diabetic patients are foot ulcers, which also involve biofilm formation; commonly caused by bacteria including, *S. aureus, Enterococcus faecalis* (*E. faecalis*), and *P. aeruginosa*.^[^ ^63^, ^64^, ^65^, ^66^ ^]^

Despite favoring pathogens during disease establishment, the ability to aggregate into biofilms confers environmental advantages: biofilm formation significantly increases antimicrobial resistance, up to 1000‐fold, due to its complex structure and metabolic influences, especially from monospecies biofilms compared to planktonic cells or polymicrobial biofilms.^[^ ^67^, ^68^ ^]^ Multidrug‐resistant biofilm‐associated bacteria contribute to treatment failures and chronic infections.^[^ ^69^, ^70^ ^]^ In hospitals, biofilms on medical devices such as catheters and implants pose serious risks, with pathogens like *S. aureus* and *Staphylococcus epidermidis* (*S*. *epidermidis*) commonly causing infections.^[^ ^71^, ^72^ ^]^

Naturally, environments inhabited by bacteria and fungi are capable of forming biofilms, as well. They are defined as communities of microorganisms of microbial cells (either single species or mixed species) adhered to the host tissue or an abiotic surface, enclosed within an extracellular polymeric substances (EPS) matrix.^[^ ^73^, ^74^ ^]^ This substance consists of conglomerates of proteins, polysaccharides, and exogenous DNA, which exhibit distinct genetic transcriptional phenotypes.^[^ ^75^, ^76^, ^77^ ^]^ These biofilms play a crucial role in protecting constituent microorganisms from external threats such as host immune systems, antimicrobials, and bacteriophages, through quorum sensing (QS) metabolic cooperation with gene expression coordinated by the community.^[^ ^78^, ^79^, ^80^ ^]^ Ultimately, the biofilm phenotype confers numerous benefits for participants such as physical and genetic factors, offering defense against the host's immune system, including suppressing phagocytosis and the complement system.^[^ ^81^ ^]^

Biofilms are responsible for ≈80% of human chronic infections, characterized by their high resistance to commonly employed treatment modalities, thereby significantly contributing to elevated mortality rates.^[^ ^82^ ^]^ These infections are associated with the use of medical devices, encompassing the upper and lower respiratory tract, valve endocarditis, bacteremia, urinary tract infections (UTIs), chronic wounds, foot ulcers, and periodontitis, all of which are pathologies associated with biofilm formation.^[^ ^15^, ^83^, ^84^ ^]^ The bacteria implicated in causing several of the aforementioned infections include *Escherichia coli* (*E*. *coli*)*, Klebsiella pneumoniae* (*K*. *pneumoniae*)*, P. aeruginosa, Acinetobacter spp., E. faecalis, Proteus mirabilis* (*P mirabilis*)*, S. aureus, and Streptococcus pneumoniae* (*S*. *pneumoniae*).^[^ ^85^ ^]^ Additionally, while a large number of studies emphasize infections caused by biofilm‐forming bacteria, studies highlighting the importance of research on biofilm‐forming fungi have been reported, as well. Investigations have revealed that most clinically significant fungi form biofilms, with *C. albicans* being the most studied. Fungal groups encompass *Fusarium, Malassezia, Trichosporon, Cryptococcus, Rhodotorula*, numerous *Candida* spp.*, Pneumocystis* sp., and endemic fungi such as *Histoplasma capsulatum* (*H*. *capsulatum*)*, Paracoccidioides brasiliensis* (*P*. *brasiliensis*)*, and Coccidioides immitis* (*C*. *immitis*).^[^ ^86^, ^87^, ^88^ ^]^

Polymicrobial biofilm formations found in infections have been demonstrated to occur between different species (e.g., *S. aureus* and *P. aeruginosa*), amongst individuals of intrinsically related or of similar genus (e.g., *P. protegens* and *P. aeruginosa*), and across different kingdoms (e.g., *S. aureus* and *C. albicans*).^[^ ^89^ ^]^ Furthermore, the occurrence of an association between *S. aureus* and *C. albicans* has been observed in cystic fibrosis, but also in diverse biofilm‐related infection contexts such as periodontitis, stomatitis, UTIs, and wound infections, characterizing the utmost important polymicrobial infections linked to biofilm.^[^ ^90^, ^91^, ^92^ ^]^ Mild infections progress to chronic stages as a result of biofilm activity, attributed to the presence of EPS, serving as a protective shield.^[^ ^67^, ^93^ ^]^

## 4 Quorum Sensing and Biofilm Dynamics

### 4.1 Biofilm Formation Mediated Quorum Sensing

During biofilm formation, whether monospecies or polymicrobial, QS regulation plays a crucial role. QS is a cell‐cell communication process dependent on population density, mediated by signaling molecules called autoinducers (AIs).^[^ ^94^, ^95^ ^]^ In Gram‐negative bacteria, signaling molecules include N‐acyl homoserine lactones (AHLs), regulating pathogenesis, synthesis, symbiosis, morphological differentiation, and secondary metabolism.^[^ ^96^, ^97^ ^]^ In Gram‐positive bacteria, signaling molecules include Type 2 autoinducers like furanosyl‐borate diester (AI‐2) and autoinducer peptide,^[^ ^38^, ^98^ ^]^ Other inducers include indole and Diffusible Signal Factor (DSF).^[^ ^99^, ^100^, ^101^ ^]^ The AHL system, also known as LuxI/R‐like QS, serves as a signaling mechanism in Gram‐negative bacteria, initially identified in *Vibrio fischeri* (*V. fischeri*) and now extensively studied and utilized.^[^ ^97^, ^102^, ^103^ ^]^ AHL is formed by several molecules, enabling the synthesis of 3‐oxo‐C6‐HSL, 3‐oxo‐C12‐HSL, and C4‐HSL in *V. fischeri* and *P. aeruginosa*.^[^ ^104^, ^105^ ^]^ The synthesis of AHL is governed by LuxI, and the LuxR‐AHL complex binds to DNA, recruiting RNA polymerase to control QS gene expression.^[^ ^106^ ^]^ When the biofilm population surpasses a certain threshold, signaling substances coordinate QS gene expression, regulating virulence factors, metabolic pathways, and biofilm development. The AHL and RhlI/R systems regulate processes such as pyocyanin and the Pel polysaccharide.^[^ ^107^, ^108^, ^109^, ^110^ ^]^

Another important QS regulation system involves autoinducer‐2 (AI‐2) borate diester signaling molecules in Gram‐positive bacteria.^[^ ^111^, ^112^ ^]^ The LuxS/AI‐2 system functions in biofilm formation, virulence factor creation, motility, and various physiological processes.^[^ ^113^, ^114^ ^]^ AI‐2 is produced from 4,5‐dihydroxy‐2,3‐pentanedione (DPD) via LuxS protein catalysis.^[^ ^115^, ^116^ ^]^ LuxS regulates the expression of the PiuA transporter in *S. pneumoniae*, increasing intracellular Fe(III) concentration and stimulating biofilm formation. LuxS also facilitates the release of extracellular DNA essential for biofilm formation in *Helicobacter pylori* (*H*. *pylori*), reducing antibiotic susceptibility in biofilms.^[^ ^117^, ^118^ ^]^ The LuxS system is present in most oral biofilms, contributing to major infections.^[^ ^119^ ^]^ A study by Wang et al.^[^ ^120^ ^]^ showed that AI‐2 supplementation elevated transcription levels of *spaP, fruA, gtfB, gtfC*, and *gtfD* genes in *S. mutans* biofilm adhesion. Deactivating the LuxS/AI‐2 system and applying synthetic precursor S‐ribosyl homocysteine, derived from methylthioadenosine nucleosidase, interferes with the LuxS/AI‐2 signaling pathway.^[^ ^121^, ^122^ ^]^ The agr system in *S. aureus* is a classic example of QS in Gram‐positive bacteria, where the autoinducing peptide regulates biofilm formation and virulence factors.^[^ ^99^, ^123^, ^124^, ^125^ ^]^ Regulation of biofilms through DSF in *S. maltophilia* was initially observed in *Xanthomonas campestris* (*X*. *campestris*).^[^ ^126^ ^]^ DSF synthesis is controlled by RpfF homolog Bcam0581, released by RpfR, which responds to genetic expression responsible for RpfC/G recognition and regulation in *B*. *cenocepacia*.^[^ ^127^, ^128^ ^]^ DSF signaling increases *Bacillus cereus* (*B*. *cereus*) susceptibility to antibiotics while inhibiting its biofilm formation.^[^ ^129^ ^]^

QS in fungal communities involves molecules like farnesol, disrupting cell membrane function and influencing morphogenesis and virulence factors in *Candida* spp. and other fungi.^[^ ^130^ ^]^ Farnesol and trisol reduce metabolic activity and biomass in polymicrobial biofilms involving bacteria such as *S. mutans*.^[^ ^131^, ^132^ ^]^ QS in polymicrobial interactions is illustrated in oral diseases, where interspecies communication affects biofilm properties.^[^ ^133^, ^134^ ^]^ Recent research highlights inter‐bacterial communication within the streptococci genus via the LuxS system, affecting biofilm properties and susceptibility to treatments. In summary, communication between different kingdoms extends beyond microorganisms within the same domain. *P. aeruginosa* utilizes 3‐oxo C12‐HSL as its primary quorum sensing molecule (QSM), suppressing filamentation and inhibiting the yeast *C. albicans*. Conversely, Candida QSM produces farnesol, capable of inhibiting two molecules of QSMs in *P. aeruginosa*. This communication is instrumental in the formation of mixed fungi‐bacteria biofilms observed in complex and resistant human infections.^[^ ^135^ ^]^ For intracellular signaling to take place, a molecule must bind to a transcriptional regulator, which subsequently regulates gene expression either positively or negatively. *S. cerevisiae* has been shown to express the elF2a kinase gene, GCN2, which is involved in stress response translation.^[^ ^136^ ^]^

**Figure 1:**
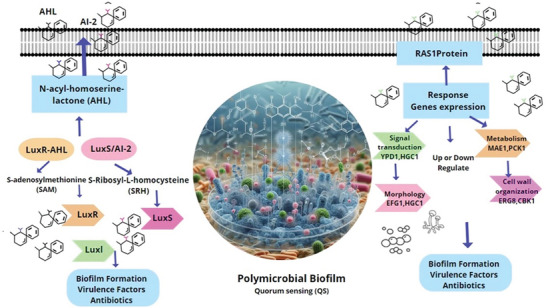
Diagram showing the interaction between the LuxS/AI‐2 quorum sensing system in bacteria and the RAS1 protein system, along with other genes involved in fungal biofilm formation, within a polymicrobial biofilm formation system.

In *C. albicans*, the C‐reactive protein Ras1, located in the plasma membrane, is an essential protein for its development and affects the formation of its biofilm. This protein governs the initiation and perpetuation of the growth of its hyphae, stimulated by GTP in the presence of farnesol, which dampens the gene expression of some genes related to morphogenesis such as (TUP, NRG1, RAS1, CYR1, EFG1, CEK1, and CHK1), to hyphal development (PDE2 and EED1), as well as drug resistance (PDR16 and INO1) respectively.^[^ ^137^, ^138^ ^]^ These signaling molecules not only influence the biofilm formation within their own species but also establish communication with other fungi of similar lineage.^[^ ^139^ ^]^ Understanding and targeting this system could represent an effective strategy to combat polymicrobial biofilm by inhibiting this signaling pathway, these mechanisms de QS are shown in **Figure** **1**.

### 4.2 Polymicrobial Interactions

PMBFs are intricate communities of microorganisms, including bacteria, fungi, and viruses, interacting across species and kingdoms within the human body.^[^ ^140^ ^]^ These biofilms colonize various sites such as oral cavities, mucosal wounds, urinary tracts, and the lungs of cystic fibrosis patients. They also attach to medical devices like catheters, prostheses, contact lenses, heart valves, and dentures.^[^ ^140^, ^141^ ^]^ Polymicrobial interactions (PMIs) are frequently implicated in diseases like cystic fibrosis (CF), where *P. aeruginosa* forms biofilms with *A. baumannii, A. fumigatus, C. cepacia, C. albicans*, and *S. aureus*. These consortia are observed in multiple diseases, indicating their adaptability in environmental defense, such as in oral cavity infections^[^ ^142^ ^]^ Similar polymicrobial compositions are found in UTIs and wound infections.^[^ ^143^, ^144^ ^]^ Immunocompromised patients are particularly susceptible to these infections, with C. albicans being a prevalent fungus in mixed bacterial‐fungal interactions.^[^ ^34^, ^145^, ^146^ ^]^

**Figure 2:**
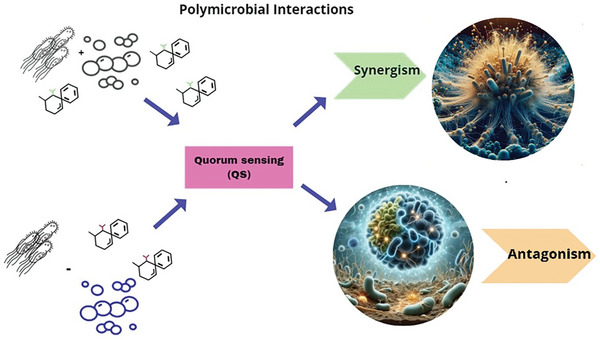
Diagram illustrates the processes of synergism and antagonism in polymicrobial interactions. Synergism refers to the collaborative interaction between microorganisms that results in an effect not achievable by any single species alone. In contrast, antagonism involves the suppression of one microorganism by another. Both processes are facilitated by quorum sensing (QS) mechanisms.

PMIs include adhesion promoted by EPS, facilitating cell proximity and intracellular interactions.^[^ ^29^ ^]^ Cell‐to‐cell communication through QS crosstalk enhances colonization, virulence, and immunoregulation by connecting pathways.^[^ ^147^ ^]^ Horizontal gene transfer (HGT) in PMBFs is complex, occurring primarily in biofilms and influenced by the genetic properties of the plasmid and host.^[^ ^148^, ^149^, ^150^ ^]^ PMBFs favor HGT due to high cell density and increased extracellular DNA (eDNA), leading to new genetic combinations and strong biofilm formation, posing a health risk.^[^ ^151^, ^152^, ^153^ ^]^ The relationships in PMBF can develop between bacteria‐bacteria and bacteria‐fungi) in a cooperative manner, with highly complex evolved mechanisms that support growth together in their environment, referring to a synergistic form (Mutualism entails both parties benefitting from the association, whereas commensalism involves one party benefiting from the other). Just as the interaction of microorganisms within the biofilm can occur fiercely due to nutrient and niche competition, establishing an antagonistic form, and these PMIs are shown in **Figure** **2**.^[^ ^154^, ^155^, ^156^, ^157^ ^]^

The host immune response plays a crucial role in infections caused by polymicrobial biofilms, widely studied for its importance in maintaining oral microflora balance.^[^ ^158^ ^]^ However, the full extent of this response and the underlying genetic mechanisms remain incompletely understood. Secretory IgA (sIgA), comprising IgA1 and IgA2 in a dimeric form, is produced in the mucous membranes and secreted in saliva to provide broad bacterial protection.^[^ ^159^ ^]^ These immunoglobulins primarily restrain excessive biofilm growth and prevent bacterial colonization, highlighting the importance of bacterial growth control over eradication. The host immune response is notably triggered by *S. mutans*, responsible for cavity formation.^[^ ^160^ ^]^

Although concrete evidence linking oral biofilms to AI‐2 and Hsp70 production is lacking, some studies suggest a potential association. Tsakmakidis et al. showed that *A. actinomycetemcomitans* and *S. oralis* could release Hsp70 in the oral mucosa as a defense mechanism. Matrix metalloproteinases (MMPs), tumor necrosis factor (TNF), and interleukin 1 are immune components activated by lipopolysaccharides (LPS) from Gram‐negative oral bacteria.^[^ ^161^ ^]^ MMPs and TNF facilitate epithelial cell destruction and extracellular matrix breakdown during inflammation, aiding tissue regeneration and promoting leukocyte infiltration.^[^ ^160^, ^162^ ^]^ Recent research indicates that epithelial cells can produce AI‐2‐like molecules involved in bacterial communication.^[^ ^163^ ^]^ Studies on bacterial genes associated with communication in *Vibrio harveyi* (*V*. *harveyi*) (VIBHAR_RS11600) suggest potential regulation of AI‐2 mimicking gene expression, present in species like *E. faecalis, S. epidermidis, S. aureus*, and *K. pneumoniae*, capable of inducing AI‐2 mimicry in epithelial cells throughout the body.

Polymicrobial dental plaque biofilms trigger the release of oral polymorphonuclear neutrophils (oPMNs) as a defense mechanism, contributing to oral cavity health.^[^ ^164^ ^]^ However, heightened oPMN production can damage oral tissues, intensify the inflammatory response, and rapidly mobilize into the bloodstream.^[^ ^165^ ^]^ Elevated PMN concentrations are observed in periodontitis patients.^[^ ^166^ ^]^ The Gram‐negative bacterium *P. gingivalis* is associated with periodontitis.^[^ ^167^ ^]^ and contributes to inflammation and bone damage through interactions with the complement system and Toll‐like receptors.^[^ ^168^, ^169^ ^]^ *P. gingivalis* orchestrates bone loss through pathobionts, commensal bacteria causing inflammation when homeostasis is disrupted.^[^ ^170^, ^171^ ^]^ Understanding and targeting the mechanisms of PMIs and immune responses is vital for developing effective treatments against biofilm‐related infections, emphasizing the need to identify therapeutic targets to restore host homeostasis.

## 5 Antimicrobial Peptides as a Promising Agent

### 5.1 Overview and Mechanisms of Action

AMPs are small, bioactive, and structurally diverse molecules found in the immune systems of various living organisms, such as fungi, mammals, and insects.^[^ ^196^, ^197^ ^]^ Although antimicrobial peptides have high selectivity, high potency, efficacy, low toxicity, and poor accumulation in tissues, they have disadvantages such as poor stability, low bioavailability, and difficulty in permeating through membranes.^[^ ^198^ ^]^ AMPs are classified by their structure and mechanism of action: the sequence of amino acids can assume three types of conformations (α‐helices, β‐sheets, and extended), while the antimicrobial mechanism can be divided into two main groups (membrane and non‐membrane targets), four subgroups (pore and non‐pore formation, cell wall target, and intracellular target), and eight models (barrel‐stave, toroidal‐pore, carpet‐like, detergent‐like, nucleic acid target, protein target, protease targeting, and cell division).^[^ ^199^ ^]^ The α‐helical AMPs are the most common in nature, and their structure allows modifications involving the addition and removal of amino acids, enhancing amphipathic properties, penetration of bacterial cell membranes, and specificity for different membrane receptors.^[^ ^200^, ^201^ ^]^

The interaction between AMPs and the bacterial cell involves several molecular and mechanical forces, which are not completely elucidated.^[^ ^202^ ^]^ However, it is widely recognized that AMPs' positively charged groups electrostatically bind to negatively charged phospholipids on the bacterial cell membrane, accumulating on its surface. As the critical concentration is reached, the peptides' hydrophobic groups insert into the membrane's lipid bilayer, altering its function and structure, leading to disarrangement, imbalance ion exchange, loss of membrane potential, disruption of cellular metabolism, and, consequently, cell death.^[^ ^203^ ^]^ In contrast, AMPs' specificity is primarily attributed to this electrostatic interaction with bacterial cells rather than host cells due to their different compositions: certain phospholipids (e.g., cardiolipin, phosphatidylglycerol, and phosphatidylserine) are present only in bacteria, while others, such as phosphatidylcholine and phosphatidylethanolamine, predominate in mammalian cells.^[^ ^204^ ^]^

Thus, the main mechanisms of action have been considered: the toroidal pore model, whereby AMPs penetrate the membrane and interact with lipids, causing toroidal pores in the membrane; the barrel‐stave model, where the peptide penetrates the membrane and creates barrel‐shaped channels; and the carpet model, where AMPs act as surfactants leading to a structural transformation of the membrane.^[^ ^205^ ^]^ To overcome properties that restrict the clinical application of antimicrobial peptides, it is necessary to enhance AMPs' stability, delivery to the infection site, and activity in formulation. Examples of strategies that have been explored include modification with natural amino acids, modification with non‐natural amino acids (including fatty acids and D‐amino acids), amino acid substitution, targeted AMP design, self‐assembly AMP therapy (AMPs self‐assemble into various nanostructures), lipopeptide AMP therapy (AMPs co‐assemble with fatty acids), combination with metal nanoparticles, and biomaterial‐based AMP delivery therapy.^[^ ^206^ ^]^ AMPs have been studied as a promising treatment strategy due to their broad‐spectrum activity and low potential for drug resistance.^[^ ^207^ ^]^ Antimicrobial peptides have also demonstrated the capacity to reverse cellular resistance to certain drugs in recent studies.^[^ ^198^ ^]^ Furthermore, AMPs have been studied for their antibiofilm properties, as they can inhibit bacterial adhesion to surfaces and reduce the expression of various genes associated with quorum sensing, matrix synthesis, or motility of bacteria in biofilms.^[^ ^208^ ^]^

### 5.2 Application Against Polymicrobial Biofilms

#### 5.2.1 AMP Mimetic Action Against In Vitro Polymicrobial Infections

Among the alternatives, AMPs have been reported as effective in treating infections caused by biofilm‐producing pathogens.^[^ ^185^ ^]^ Despite their potential, AMPs face several challenges: degradation by serum proteases, potential cytotoxicity, unfavorable physicochemical properties, short half‐life, and high production costs.^[^ ^194^ ^]^ Finding the correct concentration for AMP use is crucial. Factors such as pH, salt concentrations, and host immune responses complicate the reproducibility of in vitro tests for in vivo applications.^[^ ^209^ ^]^ For instance, a study on a tethered macrocyclic peptide (MCP) showed effective in vitro action against carbapenem‐resistant *Acinetobacter baumannii* (*A*. *baumannii*) (CRAB), but in vivo tests on rats revealed high toxicity.^[^ ^210^ ^]^

In infections by multidrug‐resistant (MDR) pathogens, combining antimicrobial drugs with AMPs can enhance their efficacy.^[^ ^211^ ^]^ Fensterseifer et al.^[^ ^212^ ^]^ analyzed an AMP (PaDBS1R6) for the treatment of Gram‐negative and Gram‐positive bacteria, showing promising results against *E. coli* and *P. aeruginosa* with minimum inhibitory concentrations (MIC) of 8 µm, and against carbapenem‐resistant *K. pneumoniae* with MIC of 16 µm. However, it did not show a synergistic effect with tetracycline or cefotaxime. For methicillin‐resistant *S. aureus* (MRSA), the MIC was 64 µm, indicating limited action. For *P. aeruginosa*, the anti‐biofilm activity of AMP showed MIC values of 8–16 µm in vitro, but the in vivo dose had to be four times higher. In a mouse model with skin infection, AMP effectiveness was not observed after four days, the critical period of infection. Factors such as peptide degradation, changes in salt concentration, stability, and host defense mechanisms may interfere with AMP‐biofilm interactions.^[^ ^213^ ^]^ Therefore, the search for new molecules and the improvement of existing ones is necessary.^[^ ^211^ ^]^

Fei et al.^[^ ^176^ ^]^ evaluated an AMP, FOTyr‐AMP, for its antimicrobial activities and biofilm elimination effect against *S. aureus* and *E. coli* in a mouse model. Despite positive in vitro and in vivo results, complete eradication of pathogenic strains was not achieved. Surviving bacterial cells can trigger resistance mechanisms, complicating treatment.^[^ ^190^ ^]^ Other studies investigated AMP LyeTx I mnΔK, derived from *Lycosa erythrognatha* spider venom, for treating *S. aureus* and *A. baumannii* in non‐surgical wounds. It demonstrated anti‐biofilm effects and inhibition of CRAB biofilm formation but faced limitations such as toxicity and degradation by serum enzymes.^[^ ^214^ ^]^ Two antifungal peptides (AFPs), NDBP‐5.7 and ToAP2, derived from scorpion venom, showed bacterial and antibiofilm activity against *C. albicans*. NDBP‐5.7 lacked anti‐biofilm activity, while ToAP2 demonstrated dose‐dependent activity but presented toxicity to mammalian cells.^[^ ^195^ ^]^ AMP LyeTx I mnΔK also showed potent effects against *C. albicans* but faced similar limitations.^[^ ^215^ ^]^

Although scarce, some AMPs have been tested as alternatives or adjuncts to antimicrobials for treating mixed infections of biofilm‐producing pathogens.^[^ ^216^ ^]^ A study evaluated tachyplesin I from the horseshoe crab and its analog (TP11A) against monomicrobial and mixed biofilms of *S. aureus* and *C. albicans*. TP11A showed promising inhibitory activity for biofilm formation but was ineffective in eradicating preformed biofilms, highlighting the difficulty of treating co‐infections with AMPs.^[^ ^186^ ^]^ Studies continue to explore the application of AMPs against mono‐ and polymicrobial biofilms, as summarized in **Table** **1**.

**Table 1:** AMP mimetic action against in vitro polymicrobial infections.

Peptide	Sequence	Bacteria Biofilm specie	Highlight	Reference
Lysostaphin	‐	*S. aureus* GFP	The bacteriocin lysostaphin was encapsulated within nanoparticles of polylactic‐co‐glycolic acid, a biodegradable copolymer with drug‐releasing capacity. This reduced the viability of bacteria in planktonic and biofilm states, making it a good option for the treatment of *S. aureus* infections.	[217]
HHC10	KRWWKWIRW‐NH_2_	MRSA	The COA‐T3 hydrogel composed of quaternized chitosan and oxidized dextran was constructed for the photosensitizer TPI‐PN and the antimicrobial peptide HHC10, presenting antibiofilm potential in the treatment of chronic infected wounds, a common condition in diabetic patients.	[218]
AMP1	VRLIVAVRIWRRG‐NH_2_	MRSA USA300, *K. pneumoniae*, *A. junii*, *P. aeruginosa*, and *E. faecalis*	The bioactive amidated antimicrobial peptides were produced by the transient line of *Nicotiana benthamiana* that expresses the mammalian enzyme peptidylglycine α‐amidating monooxygenase, presenting lethal activity against pathogenic bacteria and preventing their biofilm formation. This technology provides economic advantages and applicability for industrial use.	[219]
AMP2	VQRWLIVWRIRKG‐NH_2_			
AMP3	ILVRWIRWRIQWG‐NH_2_			
Peptides from casein (antimicrobial activity)	YYQQKPVA‐NH_2_	*S. mutans* and *P. gingivalis*	The active mixtures of antimicrobial casein peptides presented high activity in the inhibition of *S. mutants* and *P. gingivalis*, especially in the formation of biofilms, which contributes to the development of safe agents for functional foods.	[220]
	TKKTKLTEEEKNRL‐NH_2_			
	RPKHPIKHQGLPQEVLNENLLRFF‐NH_2_			
	TKVIPYVRYL‐NH_2_			
	VLNENLLR‐NH_2_			
MGD2	5,6‐carboxyfluorescein‐GLRKRLRKFFNKIKF‐NH_2_	*B. subtilis*, *S*. Typhimurium, *S. aureus*, MRSA, and *P. fluorescens*	The MGD2 peptide with different titanium binding sequences presented antimicrobial potential against different bacterial strains, especially MRSA, both in solution and when immobilized on titanium.	[221]
*N‐*salicyl‐AAn‐picolamide peptides	Sa‐GA‐Pico‐NH_2_	*P. aeruginosa* strain #14	*N*‐salicyl‐AAn‐picolamide peptides inhibited quorum sensing of pathogenic *P. aeruginosa* strain 14, presenting potential therapeutic value	[222]
	Sa‐GAG‐Pico‐NH_2_			
	Sa‐GAF‐Pico‐NH_2_			
	Sa‐GbAA‐Pico‐NH_2_			
	Sa‐G‐Gaba‐A‐Pico‐NH_2_			
P13#1	H‐NLys‐NLys‐Npet‐Npet‐NLys‐Nmpe‐Npet‐Npet‐NLys‐Npet‐Npet‐NLys‐NLys‐NH_2_	*P. aeruginosa*	P13#1 was designed to mimic cathelicidins and has bactericidal and antibiofilm activity. Furthermore, it has antimicrobial and anti‐inflammatory activities comparable to ampicillin and gentamicin without apparent toxicity.	[223]
Pln 149	YSLQMGATAIKQVKKLFKKKGG‐NH_2_	*P. gingivalis*, *S. mutans*, and *P. intermedius*	Pln 149 significantly inhibited *P. gingivalis, S. mutans* and *P. intermedius*, reducing biofilm formation and cytotoxicity. Therefore, it is considered a potential option for root canal irrigation solutions.	[224]
VTK‐LL37	CVTKLGSLChingeLL37	*E. coli* MSI001	A representative heptapeptide (VTK) was combined with the antibacterial peptide LL‐37, demonstrating bacteriostatic activity, inhibition of biofilm formation in vitro, reduced HMGB1 expression, and decreased vital organ injury in mice. Therefore, it is a good candidate for the treatment of sepsis.	[225]
P30	KNLLRRIRRKLRNKFSRSDVIKTPKIVEVN‐NH_2_	*S. aureus* ATCC 25923, CRAB KPD 205, and other five and ten Gram‐negative and Gram‐positive bacteria, respectively.	The Intestinal peptide (P30) has the ability to inhibit Gram positive and Gram negative bacteria through the formation of transmembrane pores, causing the loss of bacterial viability.	[226]
HX‐12C	FFRKVLKLIRKIWR‐NH_2_	*S. aureus* ATCC 25923	The antimicrobial peptide HX‐12C demonstrated antibiofilm ability and significant antimicrobial function in orange juice and raw pork; therefore, it is a good candidate as an antimicrobial agent in food storage.	[227]
CWR11‐AuNCs	CWFWKWWRRRRR‐ AuNCs	*S. aureus* and *A. baumannii*	Peptide‐functionalized gold nanoclusters (CWR11‐AuNCs) exhibit selective fluorescence microscopy imaging properties when bound to bacteria, enabling localization of bacteria in complex in vivo environments. They also have bactericidal and antibiofilm properties with low cytotoxicity.	[228]
P1R3	KSWKKHVVSGFFLR‐NH_2_	*S*. Typhimurium	Antimicrobial peptides P1R3 and P1C exhibited enhanced antimicrobial activities, damaging membrane functions with low cytotoxicity. Finally, P1C reduced the viability of bacteria in chicken meat at 4 °C.	[229]
P1C	KSWKKHVVSGFFLRLWVHKK‐NH_2_			
B1CTcu3	LPLLAGLAANFLPKIFCKITRK‐NH_2_	*S. aureus* and *P. aeruginosa*	The B1CTcu3 peptide, at a subminimal inhibitory concentration, presented antibiofilm activity through the membranolytic process. Furthermore, it exhibited cytotoxicity against MDA‐MB‐231 breast cancer cells with an IC50 of 25 µM.	[230]
Hp‐MAP1	AAGKVLKLLKKLL‐OH	*A. baumannii*, and *E. coli*	Both peptides exhibited in vitro activity against Gram‐negative and Gram‐positive bacteria without hemolytic effects. Furthermore, they presented interactions guided by hydrogen and salt bonds on mimetic membranes composed of anionic and neutral phospholipids, contributing to their optimization and generation of antimicrobial agents.	[231]
Hp‐MAP2	AAKKVLKLLKKLL‐OH			
Peptoid 1	(NLys‐Nspe‐Nspe)_4_‐NH_2_ and analogs	Methicillin‐susceptible *S. aureus* (MSSA) and MRSA	These peptides demonstrated efficacy against biofilm formation and detachment. Peptoid 1 can also prevent biofilm formation at a concentration of 1.6 µM. In a bioluminescent murine incision wound model of *S. aureus*, clearance of infection in treated mice occurred within 8 days, making it a good candidate for *S. aureus* wound infections.	[232]
Peptoids	Sequence‐specific oligo‐N‐substituted Glycines	*S. aureus*, *K. pneumoniae*, *A. baumannii*, *P. aeruginosa*, and *E. cloacae*	Peptoids presented good antibacterial and antibiofilm activity in vitro, both in media and under host‐mimicking conditions. They also had anti‐abscess activity in vivo.	[233]
CATHPb1	KRFKKFFRKIKKGFRKIFKKTKIFIGGTIPI‐NH_2_	*S. aureus* CMCC26003 and *V. vulnificus*	Host defense peptides exhibited effective protection for largemouth bass against bacterial infections and potent antimicrobial, antibiofilm, and immunomodulatory activities.	[234]
Cm‐CATH2	RRSRFGRFFKKVRKQLGRVLRHSRITVGGRMRF‐NH_2_			
Hc‐CATH	KFFKRLLKSVRRAVKKFRKKPRLIGLSTLL‐NH_2_			
XN2	YGNGVFSVIK‐NH_2_	*S. aureus* CICC 10384	Bacteriocin XN2 has antimicrobial activities through membrane disruption and antibiofilm. It inhibits the secretion of α‐hemolysin and regulates the QS system of *S. aureus*.	[235]
Esc(1‐21)	GIFSKLAGKKIKNLLISGLKG‐NH_2_	*E. coli* strain K12 and enterohemorrhagic *E. coli* O157:H7	Both peptides reduced biofilm formation and induced the expression of several genes related to biofilm regulation and dispersion, and participated in the stress response.	[236]
Esc(1‐18)	GIFSKLAGKKLKNLLISG‐NH_2_			
KN‐17	KWKVFKKIEKMGRNIRN‐NH_2_	*S. gordonii* and *F. nucleatum*	The KN‐17 peptide inhibits biofilm formation, has low toxicity for hBMSC cells. Furthermore, it caused RAW264.7 macrophages to transform from M1 to M2 by down‐regulating pro‐inflammatory factors and up‐regulating anti‐inflammatory factors. Its application concluded in a good candidate for prophylaxis against peri‐implant inflammation.	[237]

Peptide	Sequence	Fungi Biofilm specie	Highlight	Reference
ACP1	FLPKLGKALKKLF‐NH_2_	*C. albicans*	ACP1‐ACP1 antimicrobial peptides have fungicidal properties against *C. albicans*, they inhibit the formation of mycelium and biofilms. Therefore, it could be a promising candidate to combat fluconazole‐resistant *C. albicans* infections.	[238]
ACP2	FLPKVGKALKRLF‐NH_2_			
ACP3	FLPKIGKAIKRLF‐NH_2_			
ACP4	FLPHLGKALKHLF‐NH_2_			
ACP5	FLPKLGKAIKRLL‐NH_2_			
Myr‐B	Myr‐WRGITKVVKKV‐NH_2_	*C. albicans*, *C. tropicalis*, *and C. auris*	The Myr‐B peptide showed antifungal and antibiofilm activity and was effective in counteracting *Candida auris* and *C. albicans* infection in *Galleria mellonella* larvae, being a potential candidate for antifungal agents in human medicine.	[239]
NP339	RRRRRRRRRRRRR‐NH_2_	*C. albicans*, *A. fumigatus*, *A. flavus* and *A. niger*	NP339 targets fungal cell membranes through a charge‐charge initiated membrane interaction that has rapid fungicidal activity. Finally, NP339 is a first‐in‐class antifungal candidate for serious, invasive and neglected fungal diseases.	[240]

Peptide	Sequence	Mixed Biofilm		Reference
LfcinB (21–25)_Pal_ (P61)	RWQWRWQWR‐NH_2_	*S. salivarius*, *S. gordonii*, *A. odontolyticus*, *V. parvula*, *F. nucleatum*, *P. melaninogenica*, *P. gingivalis*, *C. albicans*, *L. paracasei*, and *C. granulosum*	The antimicrobial peptide LfcinB (21–25)Pal (P61) significantly inhibited polymicrobial biofilm, which increased during the exposure time (72 h), making it a good candidate for the treatment of localized periodontitis in patients with apnea. obstructive sleep.	[241]
Peptide amphiphiles	WWKKWKKWW‐NH_2_	*P. aeruginosa* and *S. aureus*	The research revealed that the K6 peptide demonstrated safety and efficacy in eliminating mixed biofilms of *P. aeruginosa* and *S. aureus* in a mouse model of persistent infection. These findings propose the K6 peptide as a potential candidate for antibiotic therapy. Furthermore, the exploration of additional short peptides capable of forming micelles emerges as a promising avenue in the search for new antimicrobial agents.	[242]
WMR	WGIRRILKYGKRSK‐NH2 and analogs	*C. albicans* and *A. xylosoxidans*; *C. albicans* and *S. maltophilia*	Among the synthesized peptides, the WMR‐4 peptide is capable of inhibiting mono‐ and dual‐species biofilms. It has a strong interaction with LPS and is inserted into liposomes mimicking the membranes of Gram‐negative bacteria and Candida. These results show a good candidate for the treatment of chronic lung infections in patients with cystic fibrosis.	[243]
Nisin	**I‐Dhb‐A^3^I‐Dha‐LA^7^‐Abu^8^‐PGA^11^K‐Abu^13^‐GALMGA^19^NMK‐Abu^23^‐A‐Abu^25^‐A^26^HA^28^SIHV‐Dha‐K‐OH**	*S. aureus* and *P. aeruginosa*	Nisin inhibits biofilm formation and has decreased survival of isolates. Furthermore, treatment with this bacteriocin affects the production of virulence factors such as hemolysins in *S. aureus*. Therefore, it has the potential to be an antibacterial agent in the medical and food industries.	[244]
Nile tilapia piscidin 4 (TP4)	FIHHIIGGLFSAGKAIHRLIRRRRR‐OH	*S. anginosus* and *G. vaginalis*	TP4 peptide combined with disodium EDTA and chitosan significantly decreased the number of CFU in a dose‐dependent manner. Furthermore, the TP4 microbicidal formulation significantly reduced bacterial colonization density and exhibited biocompatibility with lactobacilli and female reproductive tissues in C57BL/6 mice, making it a good candidate for topical microbicidal agents for bacterial vaginosis.	[245]

This table is separated into three topic applications: Bacteria‐, Fungi‐, and Mixed‐biofilm species. The literature collected was performed on July 14th, 2024, on SciFinder. NLys, peptoid analogue of lysine; Nmpe, N‐[2‐(4‐methoxyphenyl)ethyl]‐glycine; Npet, N‐(2‐phenylethyl)‐glycine (peptoid analogue of homophenylalanine); NPhe, peptoid analogue of Phe; Nspe, N‐1‐S‐phenylethyl; Pico, picolylamine; Sa, salicylic acid. tioether A^3^&A^7^, Abu^8^&A^11^, Abu^13^&A^19^, Abu^23^& A^26^, Abu^25^&A^28^. Dhb, dehydrobutyric acid; Dha, dehydroalanine.

#### 5.2.2 Patents Based on AMPs Against Biofilm Formation

Some patents on the use of AMPs for the inhibition and eradication of bacterial biofilms were filled up in the last few years. These patents go from the identification of new AMPs to the modification of existent peptides for better efficacy and stability. Among these, some of the most salient are those describing the identification and characterization of new, especially biofilm‐active, antimicrobial peptides; chemical or structural modifications of known AMPs to increase their resistance to proteolytic degradation and elevate their selectivity for bacterial cells; and the exploration of synergy between AMPs and other antimicrobial compounds to reach a higher efficacy in biofilm eradication. The principles described in these patents are essential in the potential to develop new therapeutic tools aimed at the elimination of drug‐resistant bacterial infections and are particularly important in both chronic and device‐associated infections. Through the provision of alternatives or complements to traditional antibiotics, AMP‐based strategies lower the selective pressure that is responsible for the development of resistance. In addition, these advances have applications beyond medicine, for instance, in the food and agricultural industries, where biofilms may cause significant problems. Overall, these patents reflect a dynamic and promising field with the potential to transform infection management and significantly improve public health and industrial safety. The patents reported according to SciFinder based on the inhibition of biofilms using peptides are shown in **Table** **2**.

**Table 2:** AMP‐Based against biofilm formation—Patent published.

Peptide	Sequence	Patent number	Bacteria Biofilm specie	Highlight	Assignee
DRGN‐1	PSKKTKPVKPKKVA‐NH_2_	WO2018022875	*P. aeruginosa*: 0.01‐10 µg mL^−1^; *S. aureus*: 0.01‐10 µg mL^−1^; Mixed biofilms.	The invention provides antimicrobial peptides for wound healing, including the discovery of DRGN‐1, a synthetic peptide derived from Komodo dragon. DRGN‐1 shows promise in treating infected wounds, including those with mixed biofilms, without harming human cells. This suggests DRGN‐1 as a potential antibiotic alternative, particularly beneficial for chronic diabetic wounds and combat injuries.	George Mason Research Foundation, Inc., United States
ATRA1A	KRAKKFFKKLK‐NH_2_				
COG1410	Ac‐AS‐Aib‐LRKL‐Aib‐KRLL‐NH_2_ and analogs	WO2024015914	*P. gingivalis*: 16–64 µg mL^−1^.	The study provides an amount of peptide compounds with antimicrobial activity, especially, against *P. gingivalis*. Also, the peptide COG1410 showed a synergism when associated with polymyxin B against *A. baumannii*	Regennova, Inc., United States
MH5C	ILGPVLGLVSDTLDDVLGIL‐COOH	WO2021154703	*P. aeruginosa*: 90 µM *E. coli*: 90 µM	The study provides a peptide conjugate comprising a polyethylene Gcol (PEG) polymer conjugated to two different antimicrobial peptides for treatment or prevention of biofilms.	University of Puerto Rico, Puerto Rico
MH5C‐Cys	ILGPVLGLVSDTLDDVLGILC‐COOH				
C0HJX7	ATCDLLSGTGANHSACAAHCLLRGNRGGYCNGKAVCVCRN‐NH_2_ and analogs	WO2018226119	*S. aureus, E. coli, P. aeruginosa, K. pneumoniae, A. baumannii*	The invention provides especially a complex of antimicrobial peptides of *Calliphora vicina* onto the anti‐biofilm activity of antimicrobials and antiseptics.	World Intellectual Property Organization.

The literature collected was performed on April 8th, 2024, on SciFinder. Aib, amino isobutyric acid

### 5.3 Challenges and Limitations

#### 5.3.1 Antimicrobial Peptide Resistance

While pathogens develop resistance to AMPs less frequently than to traditional antimicrobials,^[^ ^172^ ^]^ resistance can still occur.^[^ ^173^ ^]^ The biofilm matrix, composed of EPS, acts as a primary defense mechanism against AMPs, despite the exact role of resistance remaining unclear.^[^ ^174^ ^]^ Factors such as reduced metabolic activity of bacterial cells during biofilm formation, accumulated dead or damaged bacteria altering the matrix characteristics (pH, electrodynamics), and enzymatic degradation within the biofilm matrix contribute to this resistance.^[^ ^175^ ^]^ Incomplete elimination of pathogens in biofilm infections can select for resistant colonies. Resistance mechanisms can be intrinsic, such as efflux pumps and protease production,^[^ ^176^ ^]^ or acquired via mobile genetic elements like plasmids and transposons.^[^ ^177^ ^]^ Horizontal gene transfer of virulence and resistance genes is facilitated by conjugative mechanisms in biofilm matrices,^[^ ^51^ ^]^ complicating treatment development.^[^ ^178^ ^]^

QS signals, controlled by various virulence and resistance genes, are crucial in biofilm production. Harrington et al. conducted *ex vivo* experiments on pig lungs to simulate cystic fibrosis caused by *P. aeruginosa*, highlighting the importance of *gacA* and *pelA* genes in regulating QS and developing treatment resistance.^[^ ^179^ ^]^ However, a comprehensive understanding of the QS signaling regulation and resistance mechanisms in multispecies biofilms remains elusive.^[^ ^180^ ^]^ Mutations in genes such as *ytrA, vraG, atl*, and *namA* have been linked to stress response and tolerance in *S. aureus*.^[^ ^181^ ^]^ El Shazely et al. observed pharmacodynamic changes contributing to resistance against AMPs pexiganan and melittin, involving mutations in these genes.^[^ ^172^ ^]^ Gomes et al.^[^ ^182^ ^]^ found that pexiganan required higher concentrations to inhibit and kill co‐infections of *S. aureus* and *P. aeruginosa* compared to single‐species infections. Additionally, *S. aureus* and *P. aeruginosa* exoproducts and QS signaling interactions can hinder treatment efficacy.^[^ ^183^ ^]^ Similar resistance mechanisms are observed in fungi and other pathogens. Extracellular vesicles (EVs) play a role in biofilm formation and drug tolerance. Karkowska‐Kuleta et al. studied *C. albicans*, noting increased antifungal resistance and AMP degradation in the presence of EVs.^[^ ^184^ ^]^

In polymicrobial biofilms, pathogens can interact synergistically, antagonistically, or competitively, affecting virulence and tolerance.^[^ ^183^, ^185^ ^]^ For instance, *S. aureus* adhering to *C. albicans* hyphae in mixed biofilms complicates AMP action.^[^ ^186^ ^]^ Mixed biofilms of *C. albicans* and *S. mutans* are often linked to oral infections.^[^ ^185^, ^187^ ^]^ Li et al.^[^ ^188^ ^]^ described how these pathogens stimulate EPS matrix production, enhancing biofilm protection. Despite various reviews on resistance mechanisms,^[^ ^173^, ^175^, ^189^, ^190^, ^191^ ^]^ the interactions and resistance mechanisms in polymicrobial biofilms remain unclear.^[^ ^183^, ^192^ ^]^ Addressing these challenges requires strategies to enhance AMP stability and bioavailability, such as combining AMPs with antimicrobials, using nanotechnology, and modifying peptide structures.^[^ ^182^, ^193^, ^194^, ^195^ ^]^

#### 5.3.2 AMPs and Synergism Interactions

Polymicrobial synergy refers to the collaborative interactions among microorganisms, resulting in effects that cannot be achieved by any single species alone.^[^ ^34^, ^154^, ^155^, ^253^ ^]^ This process involves physical combinations, metabolic collaboration through cross‐feeding, enzymatic enhancement, quorum sensing, gene transfer, enhanced growth, antimicrobial tolerance, virulence, and sustained high EPS production.^[^ ^141^, ^254^, ^255^, ^256^, ^257^ ^]^ The initial phase of polymicrobial biofilm formation and subsequent infection involves microorganism adhesion to surfaces, which differs in multispecies interactions compared to single‐species biofilm formation.^[^ ^258^ ^]^ Streptococci, common oral colonizers, adhere to salivary films or coaggregate with other microorganisms using serine‐rich cell wall proteins (SRRPs). For instance, *S. parasanguinis* expresses SrpA, while *S. sanguinis* and *S. cristatus* produce SrpA, and *S. salivarius* exhibits Srp A, B, and C.^[^ ^259^ ^]^ These proteins transport bacteria to the oral cavity by binding to the salivary film, exemplified by the interaction between *S. gordonii* and *Veillonella* spp., facilitated by *S. gordonii* binding to the sialic acid of mucin at α‐2,3.^[^ ^260^, ^261^ ^]^ *S. gordonii* also encodes genes sspA and sspB,^[^ ^262^ ^]^ with SspB mediating the adhesion to *C. albicans* by binding to the hyphal‐specific glycoprotein Als3p, enhancing biofilm formation, which is further supported by *S. intermedius*.^[^ ^263^ ^]^ S. mutans promotes EPS production and the colonization of lactobacilli through glycosyltransferases (Gtfs), which cleave sucrose to produce extracellular glucans.^[^ ^265^ ^]^

In multispecies biofilms, QS facilitates biofilm formation through specific co‐aggregation interactions. Gram‐negative species like *P. aeruginosa* use AHL systems, while peptides regulate QS in *S. aureus* and other Gram‐positive bacteria.^[^ ^49^, ^266^, ^267^ ^]^ Co‐metabolism and syntrophy within biofilms involve one species consuming the byproducts of another. Hansen et al. described how *P. putida* and *Acinetobacter* spp. coexist by metabolically interacting; *Acinetobacter* spp. metabolizes benzyl alcohol into benzoate, used by *P. putida*, enhancing biofilm metabolic output and antibiotic resistance.^[^ ^268^, ^269^ ^]^

Fungal‐bacterial interactions have been documented by Medina‐Alarcón et al. (2021). Co‐infections between *M*. *tuberculosis* and *P. brasiliensis* primarily affect immunocompromised patients and account for about 20% of cases in South America, especially Brazil. These pathogens form stronger biofilms together than individually, likely due to genetic transfer facilitating their interaction.^[^ ^270^ ^]^ In oral biofilms, *S*. *mutans* shows a significantly higher transformation rate within biofilms compared to planktonic states, enhancing bacterial adhesion and biofilm formation.^[^ ^271^ ^]^ Other Streptococcus species, including *S. mitis*, *S. oralis*, and *S. pseudopneumoniae*, also engage in gene transfer, influencing their evolutionary dynamics.^[^ ^272^, ^273^ ^]^ The co‐isolation of *C. albicans* and MRSA from host tissues indicates a synergistic interaction where *C. albicans* protects MRSA from vancomycin treatment by exogenous supplementation of β‐1,3‐glucan, suggesting a protective effect.^[^ ^274^, ^275^ ^]^ Multispecies biofilms serve as platforms for collaboration among evolving species, leading to enhanced resistance and functionality, as described by the ‘Red Queen Hypothesis’. This hypothesis posits that competition and antagonistic interactions drive the co‐evolution of species, resulting in interdependent relationships.^[^ ^276^, ^277^ ^]^

Some challenges encountered during resistance to AMPs and/or during polymicrobial synergism could be overcome by employing multi‐attack combination techniques such as combination therapy, or the use of AMP‐antibiotic synergism. Using antimicrobials in combination with AMPs has shown enhanced anti‐pathogen effects. Ceftazidime and piperacillin demonstrated synergistic effects with AMPs containing Trp residues against *P. aeruginosa* biofilms, reducing virulence and inhibiting efflux pump gene expression, thus enhancing antibiotic activity at lower doses.^[^ ^278^ ^]^ The combination of ToAP2 and NDBP‐5.7 AMPs with antifungals like amphotericin B and fluconazole showed synergistic effects against *C. albicans*, suggesting promising treatment options.^[^ ^195^ ^]^ The synergistic interaction of nisin and pexiganan AMPs was effective against *S. aureus* and *P. aeruginosa* in a 3D collagen matrix model simulating diabetic foot wound biofilms. A guar gum biogel coupled with AMPs showed potential for topical use in treating polymicrobial infections.^[^ ^182^ ^]^ The combination of Pom‐derived peptides with fluconazole demonstrated promising anti‐biofilm activity against *C. albicans*.^[^ ^279^ ^]^ In another study, an AMP (W379) combined with a monoclonal antibody (anti‐PBP2a) delivered via a microneedle patch showed promising results in inhibiting MRSA biofilms.^[^ ^280^ ^]^ Fan et al. proposed using fluorophore‐conjugated photosensitizers coupled with AMPs (TPI‐CysHHC10), which successfully inhibited and destroyed mature MRSA biofilms.^[^ ^281^ ^]^ Recent advances on the synergistic effect between antibiotics and peptides are shown in **Table** **3**.

**Table 3:** AMP‐Based against mono‐specie biofilm formation—journal published.

Peptide (sequence)	Antibiotic	Bacteria/Fungus % biofilm Inhibition	Reference
nisin from *Lactococcus lactis* I‐Dhb‐A^3^I‐Dha‐LA^7^‐Abu^8^‐PGA^11^K‐Abu^13^‐GALMGA^19^NMK‐Abu^23^‐A‐Abu^25^‐A^26^HA^28^SIHV‐Dha‐K‐OH	Oxacillin sodium monohydrate	Inhibition of methicillin (oxacillin)‐resistant *S. aureus* and methicillin‐resistant *S. epidermidis* biofilm in a rage of 90‐75% at 1xMIC	[282]
RQ18 RALRKALKAWRKLAKKLQ‐NH_2_	Anfotericin B	Inhibition of *C. tropicalis* ATCC 750 biofilm in 87,7% at 1 x MIC	[283]
Ana‐9 Ac‐R^6^‐d‐Nal^7^‐R^8^‐W^9^‐d‐Nal^10^‐K^11^‐F^12^‐V^13^‐CONH_2_	Oxacilin	Inhibition of methicillin (oxacillin)‐resistant *S. aureus* biofilm in 87%	[284]
Melittin GIGAVLKVLTTGLPALISWIKRKRQQ‐NH_2_	vancomycin	Inhibition of methicillin (oxacillin)‐resistant *S. aureus* biofilm at 64±2.5 µg mL^−1^	[285]
LL‐37 LLGDFFRKSKEKIGKEFKRIVQRIKDFLRNLVPRTES	Polymyxin B	Reducion of *E. coli* MG1655 and *P. aeruginosa* PAO1 biofilm at 2 µg mL^−1^ Polymyxin B + 16 µg mL^−1^ LL‐37	[286, 287]
Melittin GIGAVLKVLTTGLPALISWIKRKRQQ‐NH_2_	Oxacilin	Inhibition of methicillin (oxacillin)‐resistant *S. aureus* biofilm in 65% at 0.75xMIC	[288]
CRAMP‐34 GLLRKGGEKIGEKLKKIGQKIKNFFQKLVPQPEQ	Vancomycin	Reduce of 2 log_10_ CFU mL^−1^ in *P. aeruginosa* PAO1 at 0.25x MIC CRAMP‐34 + 0.25 x MIC vancomycin	[289]
TAT‐RasGAP_317‐326_ DTRLNTVWMWGGRRRQRRKKRG	Gentamicin	Combining TAT‐RasGAP317‐326 + gentamicin does not reduce *A. baumannii* biofilm biomass formation effectivy	[290]
Melittin GIGAVLKVLTTGLPALISWIKRKRQQ‐NH_2_	Rifampicin	Minimum Biofilm Preventive Concentration of 0.039 µg mL^−1^ of melittin and 256 µg mL^−1^ of rifampicin in methicillin‐resistant *S. epidermidis*	[291]
Bacteriocin XJS01 produced by *Lactobacillus salivarius*	Phenyllactic acid	Reduction of *S. flexneri* biofilm at 0.5xMIC phenyllactic acid + 0.5 xMIC XJS01	[292]
P255 Ac‐RKKWFW‐NH_2_ P256 Ac‐WKKWFR‐NH_2_	Amphotericin B	Reduction of *C. albicans* biofilm in 84.5% and 73.7% at 0.25 xMIC amphotericin B + 0.5 xMIC P255 and 0.25 xMIC of amphotericin B + 0.5 xMIC P256, respectively	[293]
Polymyxin B	Azithromycin	Fractional *A. baumannii* biofilm inhibitory concentration index range 0.19–0.31	[294]
*Mo*‐CBP3‐PepIII NIQPPCRCC	Ciprofloxacin	Inhibition of *S. aureus* ATCC 25923 biofilm in 76% at 3.1 µg mL^−1^ *Mo*‐CBP3‐PepIII + 0.2 µg mL^−1^ ciproflozacin	[295, 296]
Ceragenin CSA‐131	Sulfamethoxazole	Inhibition of *S. maltophilia* biofilm formation in 90% at 5 µg mL^−1^ CSA‐131+ 5 µg mL^−1^ Sulfamethoxazole	[297]
KR‐12‐a5 KRIVKLILKWLR‐NH_2_	Epigallocatechin gallate	reduced *E. faecalis* (ATCC 51,299 biofilm (‐5.52 log CFU mL^−1^) in EGCG 0.6 mg mL^−1^ and KR‐12‐a5 0.3 mg mL^−1^	[298]
LyeTx I‐b IWLTALKFLGKNLGKLAKQQCAKL‐NH_2_	Maleimide mPEG	LyeTx I‐bPEG reduced the Carbapenem‐resistant *A. baumannii* biofilm by 33 ± 4% at 16 µM	[299]
Pt5‐1c SRMKKWAKIIEKWRKWHKKRWLAHHSATK	Oxacillin	Inhibition of *S. aureus* USA500 biofilm formation in 99% at 0.031×MIC Pt5‐1c + 0.125 ×MIC oxacillin	[300]
CRAMP GLLRKGGEKIGEKLKKIGQKIKNFFQKLVPQPEQ	Colistin	Reduction of *P. aeruginosa* PAO1viable biofilm bacteria in 2 log_10_ at 0.25 xMIC CRAMP+ 0.25 xMIC colistin	[301]
HPRP‐A1 KLKKLFSKLWNWK‐OH HPRP‐A2 FKKLKKLFKLWNWK‐OH (D‐amino acids)	Chlorhexidine acetate (CHA)	The minimal biofilm inhibitory concentration in *K. pneumoniae* of HPRP‐A1 1xMIC + CHA 1xMIC is 0.5 µM and 1xMIC HPRP‐A2 + CHA 0.25xMIC is 0.25 µM	[302]
r(P)ApoBLA PHVALKPGKLKFIIPSPKRPVKLLSGGNTLHLVSTTKTA‐NH_2_	gentamicin or tetracycline	*E. aerogens*, *E. coli, S. aureus, S. epidermidis*, *P. mirabilis*, and *P. vulgari*	[303]

^a)^ Polymicrobial biofilm (multi‐species of bacteria). tioether A^3^&A^7^, Abu^8^&A^11^, Abu^13^&A^19^, Abu^23^& A^26^, Abu^25^&A^28^. Dhb, dehydrobutyric acid; Dha, dehydroalanine;

The literature collected was performed on April 10th, 2024, on SciFinder.

#### 5.3.3 Salt Sensitivity and its Implications

AMPs rely heavily on their interaction with bacterial membranes, which are typically negatively charged and this interaction is facilitated by positively charged amino acid residues in AMPs, which are attracted to negative components of bacterial cell membranes, leading to membrane disruption and bacterial cell death^[^ ^304^ ^]^ However, in environments with high ionic strength, such as physiological fluids containing salts like sodium chloride, the electrostatic interactions between AMPs and bacterial membranes are weakened. Salts can neutralize the positive charges on AMPs, reducing their ability to effectively bind to the bacterial surface.^[^ ^305^ ^]^ This phenomenon, known as “salt sensitivity,” can significantly decrease the potency of AMPs, making them less effective in physiological or infection‐relevant environments, such as blood, mucosa, or wounds.^[^ ^306^ ^]^

This salt sensitivity presents a major challenge for the use of AMPs as therapeutic agents, since human physiological fluids, including blood and interstitial fluids, contain high concentrations of salt, which considerably compromises the antimicrobial activity of many AMPs in these environments. AMPs that perform well under laboratory conditions with low salinity may not exhibit the same level of effectiveness in the human body, where the ionic environment is more complex.^[^ ^307^ ^]^ This limits their applications, since they can only be used in environments with low or controlled ionic strength, thus restricting their use in systemic treatments for bacterial infections. Therefore, it is necessary to optimize and structurally modify AMPs to improve their salt tolerance and ensure their efficacy in real clinical settings.^[^ ^308^ ^]^

To address the salt sensitivity of AMPs, various strategies have been developed. One of them is a structural modification, which can include increasing hydrophobicity by adding nonpolar residues, cyclizing peptides to reduce their conformational flexibility, or incorporating unnatural amino acids that maintain their charge or structural integrity despite ionic fluctuations.^[^ ^309^ ^]^ Another strategy is the design of hybrid molecules, combining AMPs with other compounds such as antibiotics or nanoparticles, which can improve their stability and efficacy, even under high salinity conditions (Section 6.1). Finally, salt‐insensitive AMPs are being developed by optimizing peptides that intrinsically show low sensitivity to salt concentrations, allowing for more effective clinical candidates.^[^ ^310^ ^]^ Some modified AMPs, such as cathelicidin derivatives, synthetic peptides or marine peptides, have been shown to maintain their antimicrobial efficacy even in physiological ionic environments, serving as models for the development of next‐generation peptides with greater therapeutic potential.^[^ ^307^ ^]^

## 6 Future Directions

### 6.1 Nanotechnology, Future Directions, and Expert Opinion

To counteract the resistance of bacteria and fungi to drugs, several drug delivery strategies have been reported against MDR pathogens. Nanotechnology is a key tool for the transport and application of various unstable or sensitive molecules that could have their action compromised by external factors. Many of these systems involve the use of polymers, lipids, metals, or inert materials, which can be simple (known as nanocarriers) or modified (referred to as nanotarget carriers). AMPs in nanotechnology can be used strategically in several ways. AMPs can be encapsulated, which is the most commonly used method when antimicrobial or biofilm inhibition values are promising. There is also the possibility of including AMPs as part of self‐organized structures within the nanosystem, known as self‐assembly, due to the amphipathic nature of AMPs. Additionally, they can be conjugated to the nanostructure for targeted delivery.

**Figure 3:**
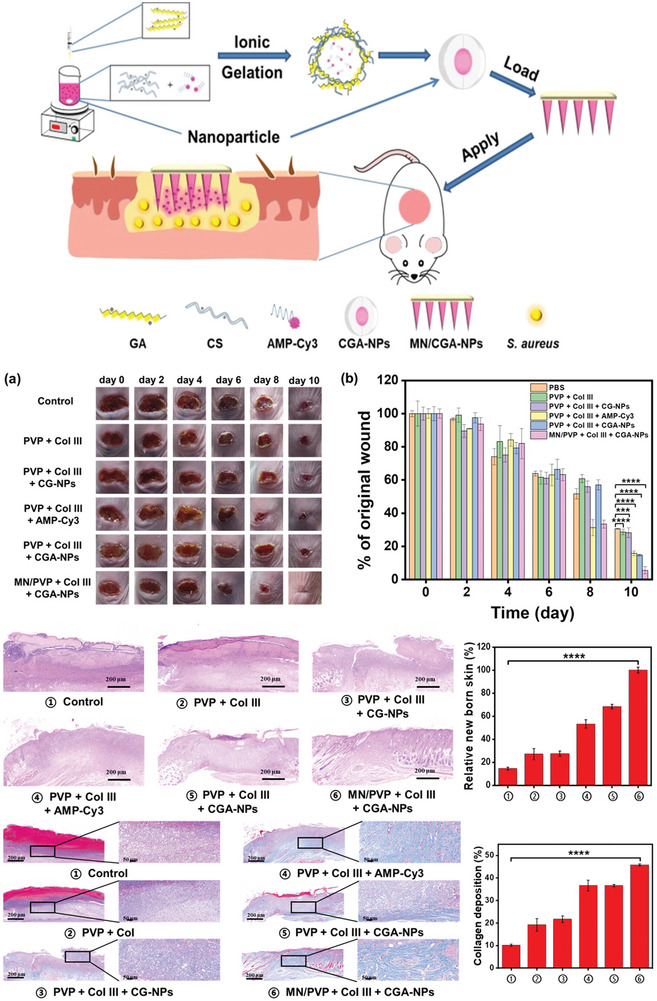
Preparation and Application of MN/CGA‐NPs. Wound healing in mice after 10 days of treatment, showing wound images (a) and wound area quantification (b) for infected mice receiving different treatments. H&E staining images and quantitative data of new skin tissue from *S*. *aureus*‐infected mice. Masson's trichrome staining images and quantitative data of collagen deposition in skin tissue from *S. aureus*‐infected mice. Reproduced (adapted) with permission.^[^ ^311^ ^]^ Copyright 2023, American Chemical Society.

Some studies were reported to indicate the promising activity of these nanostructured systems against biofilm‐forming bacteria. Recently, Wang et al.,^[^ ^311^ ^]^ reported the development of microneedle patches containing AMP and recombinant type III humanized collagen (Col III) for the slow and controlled release of these compounds, aiming to eliminate bacteria in deep wound tissue and promote healing. These AMP dissolved upon penetrating infected skin and biofilms formed by *S. aureus*, releasing the CGA‐NPs. It responded to the infected microenvironment to effectively kill bacteria, while Col III promoted wound healing. This approach represents a strategy for designing and applying a microneedle‐based antimicrobial drug delivery system, as fully explained in **Figure** **3**. Some strategies that involve the use of AMPs against biofilms can be simultaneously employed in micro‐structured systems to prevent the dissemination of bacteria, thereby preventing systemic infection and avoiding reinfection.^[^ ^312^ ^]^

**Figure 4:**
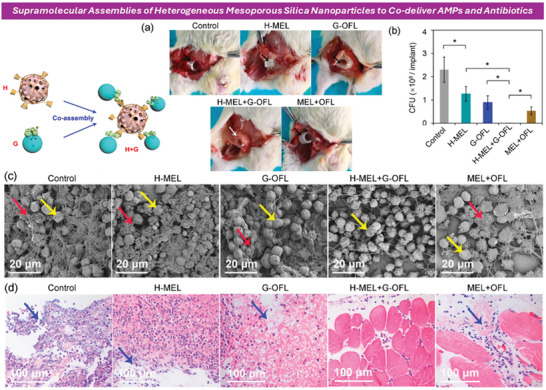
In vivo antibiofilm activity of MSNs and coassemblies. a) Images of biofilm‐colonized implants in mice treated with drug‐loaded single MSNs, coassemblies, or free drugs. Black arrows indicate implants failing to adhere to host tissue; white arrow indicates successful adhesion. b) Quantification of bacterial cells on biofilm‐colonized implants. c) SEM images of implant inner surfaces, with red arrows highlighting bacterial cells and yellow arrows indicating host cells. d) Histopathological images of tissues at implant sites, with blue arrows indicating damaged host muscle tissues suffering from inflammation. Reproduced (adapted) with permission.^[^ ^313^ ^]^ Copyright 2020, American Chemical Society.

In a study reported by Yu et al.,^[^ ^313^ ^]^ a magnetic supramolecular nanoplatform sensitive to multiple stimuli was designed for the co‐administration of high and low‐molecular‐weight drugs, achieving the synergistic eradication of pathogenic biofilms. Under the stimulation of pathogenic cells and heating by an alternating magnetic field (AMF), the supramolecular coassemblies efficiently released the high‐molecular‐weight antimicrobial peptide melittin (MEL) and the low‐molecular‐weight antibiotic ofloxacin (OFL). Compared to free drugs (MEL and OFL) or unassembled MSNs (H or G), drug‐loaded H+G coassemblies (H‐MEL+G‐OFL) demonstrated a significantly higher ability to eradicate biofilms, completely eliminating the biomass and killing pathogenic cells, without apparent toxicity to mammalian cells. Furthermore, an in vivo implantation model demonstrated that the coassemblies effectively eradicated pathogenic biofilms from the implants, preventing host tissue damage and inflammation. Therefore, these multistimuli‐responsive nanocarriers offer a promising solution for overcoming biofilm‐associated infections during infection treatment (**Figure** **4**).

In the study by Han et al.,^[^ ^314^ ^]^ supramolecular nanoparticles sensitive to matrix metalloproteinase (MMP), called MMP‐S nanoparticles, were developed to enhance the photodynamic antibacterial effect against biofilm‐associated bacterial keratitis. The nanoparticles had a negatively charged glutamic acid‐rich peptide coating, preventing adhesion to the normal ocular surface or healthy corneal cells, thereby improving tear retention. The exposed cationic peptides facilitated nanoparticle penetration and accumulation in the biofilms and binding to the Gram‐negative bacteria *P. aeruginosa*, enhancing the photodynamic antibacterial effect. Additionally, the inflammatory response in the cornea was significantly inhibited, preventing further corneal tissue damage.

Recent advances in molecular design, self‐assembly, and AMP‐based nanomaterials offer promising solutions to AMP therapy challenges.^[^ ^315^, ^316^ ^]^ Nanoparticles can protect AMPs from degradation and maintain their stability until they reach the target site.^[^ ^316^, ^317^ ^]^ Xiao et al.^[^ ^318^ ^]^ developed a polysaccharide and elastin‐like polypeptide system capable of forming nanoparticles that self‐assemble based on temperature. Additionally, hydrogels with nanofiber‐coated peptides have shown strong antimicrobial activity and immunoregulatory properties, enhancing wound healing.^[^ ^319^ ^]^

**Figure 5:**
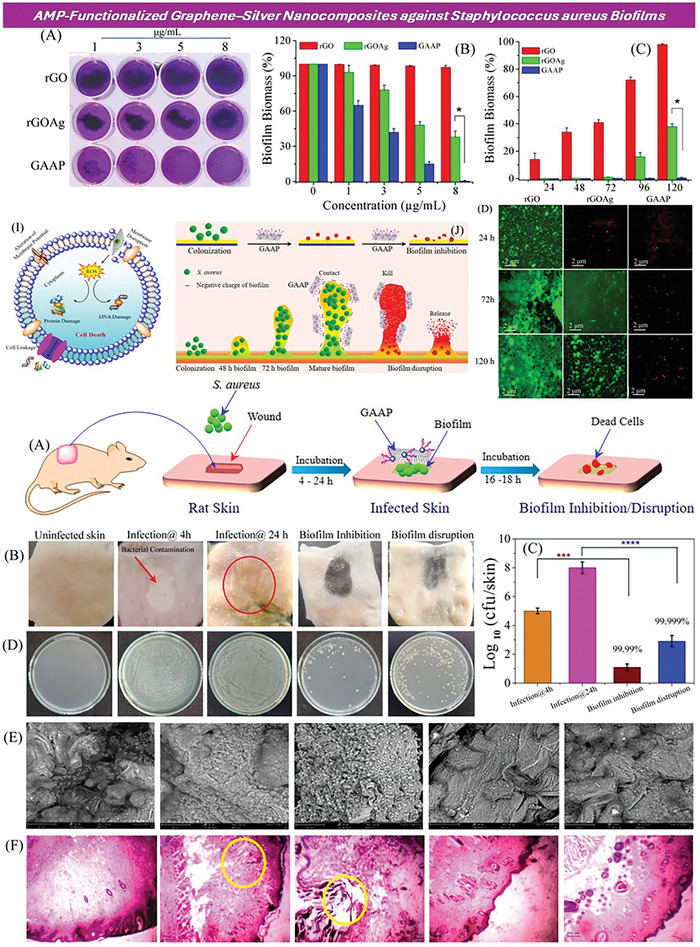
UP) The crystal violet assay (A) was used to measure biofilm formation on rGO, rGOAg, and GAAP after incubating *S. aureus* for 120 h. The biofilm inhibition activity of rGO, rGOAg, and GAAP against *S*. *aureus* was assessed in a concentration (B)‐ and time (C)‐dependent manner. Fluorescence microscopy images (D) displayed the formation of *S*. *aureus* biofilms upon interaction with rGO, rGOAg, and GAAP at different time intervals (24, 72, and 120 h); the scale bar was 2 µm. Live cells were shown in green‐fluorescent color, while dead cells with compromised cell membranes were depicted in red fluorescent color. (DOWN) A schematic representation (A) illustrated the antibiofouling activity of GAAP in an *ex vivo* rat skin infection model. Digital images (B) showed uninfected control skin (first panel), *S. aureus* infected skin after 4 h (second panel), *S*. *aureus* infected skin after 24 h (biofilm formation), and biofilm inhibition (fourth panel) and disruption (last panel) by GAAP. The bacterial count (C) was measured using the agar plate dilution method before and after a single‐dose treatment with GAAP (10 µg mL^−1^). Quantitative measurement (D) of the bacterial count before and after GAAP treatment was provided. SEM images (E) and histological analysis (F) were included for the uninfected control (first panel), skin tissue after 4 h of infection (second panel), skin tissue after 24 h of infection (biofilm formation), and GAAP‐treated skin tissue after 4 h of infection (biofilm inhibition, fourth panel) and 24 h of infection (biofilm disruption, last panel). Reproduced (adapted) with permission.^[^ ^320^ ^]^ Copyright 2021 American Chemical Society.

Parandhaman et al.^[^ ^320^ ^]^ reported an eco‐friendly strategy for synthesizing and functionalizing graphene–silver (rGOAg) nanocomposites with an AMP to treat *S. aureus* infections. The *ex vivo* rat skin disinfection model further demonstrated GAAP's effectiveness in eliminating biofilm formation and disrupting the *S. aureus* biofilm. The results shown in **Figure** **5** provided a general approach for designing functional nanografted composite materials to disrupt mature biofilms and offered a promising strategy for treating bacterial infections.

**Figure 6:**
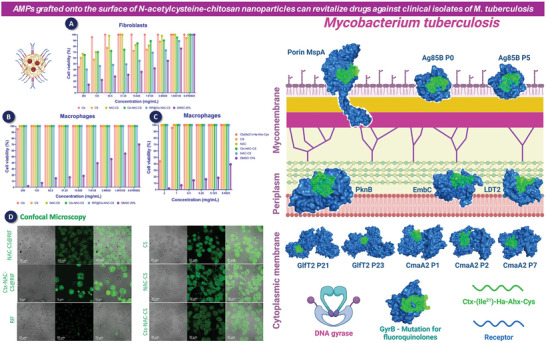
(Left) A) Cellular viability of nano‐conjugated chitosan in macrophages after 24 h of exposure. B) Cellular viability of nano‐conjugated chitosan in fibroblasts after 24 h of exposure. C) Cellular viability of conjugated chitosan polymers in macrophages after 24 h of exposure. D) Confocal analysis of internalization of FITC‐NPs in macrophages after 24 h of incubation. (Right) Molecular docking – peptide receptor interaction in *M. tuberculosis*. The bacterial receptors (Ag85B, LTD2, GyrB, EmbC, GlfT2, Porin MspA, PknB, and CmaA2) are represented in blue, and the AMP Ctx(Ile^21^)‐Ha‐Ahx‐Cys (Ctx) is represented in green. Reproduced (adapted) with permission.^[^ ^201^ ^]^ Copyright 2023, Elsevier.

While the examples presented in this section (**Table** **4** and Figures 3 and 4) are based on reports used against mono‐ or unicellular biofilms, as there are no reports of AMP applications against polymicrobial biofilms in mice to our knowledge, we highlight that their application could be extremely promising against polymicrobial biofilms. Using suitable AMPs for future research, as outlined in the application tables shown in previous sections, could yield significant results. Nanosystems can be beneficial during transport and biomedical applications against resistance, especially in biofilm formation. Modifications to the nanostructure could be crucial for eliminating various response mechanisms. Primo et al.^[^ ^201^ ^]^ demonstrated the ability to reverse rifampicin resistance in clinical isolates using an antimicrobial peptide conjugated on the surface of *N*‐acetylcysteine‐chitosan nanoparticles through disulfide bridges. Potential targets for these nanoparticles were corroborated through molecular docking using specific receptors of *M. tuberculosis*. These advances have opened new horizons in nanobiotechnology and its potential response to the era of resistance (**Figure** **6**).

**Table 4:** AMP in nanosystem against the formation of monobacterial biofilms—published journal. The literature collection was performed on April 17, 2024 on SciFinder.

Peptide (sequence)	Macromolecules	Bacteria/Fungus % biofilm Inhibition	In vivo	Nanostructure	Reference
LYS (Recombinant lysostaphin from *Staphylococcus simulans)*	Poly(lactic‐*co*‐glycolic acid) (PLGA)	Inhibition of *S. aureus* biofilm formation at 200 µg mL^−1^	No	Emulsion	[217]
Nisin I‐Dhb‐A^3^I‐Dha‐LA^7^‐Abu^8^‐PGA^11^K‐Abu^13^‐GALMGA^19^NMK‐Abu^23^‐A‐Abu^25^‐A^26^HA^28^SIHV‐Dha‐K‐OH	Thiolated Chitosan	Inhibition of *S. aureus* and *L. monocytogenes* biofilm formation in 100%	No	nisin and selenium encapsulated in thiolated chitosan nanoparticles	[321]
HHC36 KRWWKWWRR	Titanium	Inhibition of *S. aureus, E. coli*, *P. aeruginosa* and MRSA biofilm formation in 22%,50%,26% and 44% respectivity	Yes	diselenide‐bridged mesoporous silica nanoparticles	[322]
Ura56‐PEG Sequence no reported	HAuCl_4_	Inhibition of *S. aureus* biofilm formation in 80% at 0.5 µM (4xMIC)	Yes	Gold nanoparticles	[323]
PAMAM‐PLL Dendritic structure with polyamide amine (PAMAM) as core and polylysine (PLL) as branched chain	Gold	Inhibition of MRSA biofilm formation in 93% at 62.5 µg mL^−1^	No	Gold nanoparticles	[324]
NZ2114 (GFGCNGPWNEDDLRCHNHCKSIKGYKGGYCAKGGFVCKCY)	PLGA	Inhibition of *S.epidermidis* mature biofilm formation in 100% at 32xMIC	No	Emulsion	[325]
CR CR‐NH_2_	HAuCl_4_	Inhibition of *S. aureus* at 4x MIC	Yes	Gold nanoparticles	[326]
F6P6 MSPSILKHWSRQGGGKKLLGLLLKLKLK	EPV gut‐targeted engineering particle vaccine	Inhibition of *Clostridium perfringens* in 70% at 256 µg mL^−1^	Yes	Engineering particle vaccine	[327]
AMPNP N3‐CKR‐12	Polycarbonate membrane	MRSA biofilm ablation of 41%	Yes	Liposome	[328]
HSP KKKVVVHKVVVKK	ZnCl2	Inhibition of *MRSA mature* biofilm formation in 80% with nanoparticles+phtodynamix therapy	Yes	Photodynamic nanoparticles	[329]

tioether A^3^&A^7^, Abu^8^&A^11^, Abu^13^&A^19^, Abu^23^& A^26^, Abu^25^&A^28^. Dhb, dehydrobutyric acid; Dha, dehydroalanine.

### 6.2 Bioconjugations

The conjugation or modification of the *N*‐terminal of the peptide with other macromolecules has emerged as a promising strategy in the fight against bacterial resistance. This approach involves attaching functional macromolecules, such as other peptides, proteins, or polymers, to the *N*‐terminal end of a compound, thereby enhancing its antimicrobial properties. By modifying the *N*‐terminal, it is possible to improve the stability, specificity, and efficacy of the therapeutic agents. Such modifications can disrupt bacterial cell walls, inhibit essential bacterial enzymes, or enhance the immune response against pathogens. Additionally, the *N*‐terminal conjugation can be tailored to target specific bacterial strains, reducing the likelihood of resistance development. This innovative strategy holds significant potential in developing new treatments for resistant bacterial infections, addressing a critical need in modern medicine. Some strategies are used during peptide structural modification such as siderophore peptide, antibiotic‐peptide, glyco‐ and lipidation, metal peptide conjugates, and peptidomimetics. **Table** **5** shows the recent applications of structural modification that were successful in biofilm inhibition as well as the elimination of these pathogens.

**Table 5:** AMP conjugation‐Based against monobacteria biofilm formation—journal published. The literature collection was performed on April 16th, 2024, on SciFinder.

Peptide (sequence)	Conjugates	Bacteria/Fungus % biofilm Inhibition	Reference
G3 GIIKKIIKKIIKKI‐ NH_2_	Ser‐Galactose and Caprylic acid	Inhibition of *P. aeruginosa* biofilm in 71% at 2xMIC	[330]
CGA‐N9 RILSILRHQ	*N*‐octanoic	Inhibition of *C. albicans* biofilm formation in 50.5 µg mL^−1^	[331]
FKKL	G2 PAMAM dendrimer	Inhibition of *E. coli* biofilm formation ofapproximately 98%	[332]
III5 Sequence no reported	2‐heptamine	Inhibition of MRSA biofilms in 80% at 128 µg mL^−1^	[333]
E6 RRWRIVVIRVRRC‐NH_2_	Polydopamine	Inhibition of *P. aeruginosa* biofilms in 98.4%	[334]
C10‐KR8d KRIWQRIK	C10 Acyl‐chain	Inhibition of *S. aureus* USA300 LAC biofilm in 100%	[335]
Mur‐B WRGITKVVKKV	myristoyl moiety	Inhibition of *C. albicans*, *C*. *tropicalis*, and *C*. *auris* isolates at128 µg mL^−1^	[239]
C12‐DK5 IKKILS*K*IKKLL‐NH_2_	CH_3_(CH_2_)_10_C(O)‐	Inhibitory concentration that reduced *S. aureus* and *C. albicans* biofilm development at 6.25 µg mL^−1^	[336]
LyeTxI‐b IWLTALKFLGKNLGKLAKQQKAKL	Acetyl	Inhibition of *S. aureus* biofilm at 46 µmol/L	[337]
CT9W_1000_ Sequence no reported	PEG	Inhibition of *P. aeruginosa* PAO1 biofilms at 2×MIC	[338]
PEG6‐IDR1018 VRLIVAVRIWRR‐NH_2_	PEG	Inhibition of MRSA0017 biofilms in 90% at 64 µg mL^−1^	[339]
L163 Ac‐FLPLIGGLLKGLL‐NH_2_	Acetyl	It inhibited the formation of MDR *S. aureus*, *Streptococcus suis*, and *L*. *monocytogenes* biofilms.	[331]

## 7 Final Considerations

Due there is a deficit in finding new anti‐biofilm and antibacterial agents, this review demonstrates that AMPs are excellent candidates, particularly for inhibiting polymicrobial biofilms. AMPs hold great promise as alternatives against a range of pathogens, however, through this review, we aim to emphasize medicinal chemists, pharmaceutical companies, and current researchers as well, to the potential of AMPs, as few reports have progressed to the preclinical phase against polymicrobial biofilms. We urge that research global efforts to recognize that the battle against bacterial and fungal resistance is only just beginning. Polymicrobial biofilms will pose the greatest threat in this new era of resistance, and drastic measures must be taken to prepare and mitigate this challenge. Therefore, as the research on AMPs advances, we must establish best practices against pathogens and polymicrobial biofilm formations.

## Conflict of Interest

The authors declare no conflict of interest.
